# Spread and change in stress resistance of Shiga toxin-producing *Escherichia coli* O157 on fungal colonies

**DOI:** 10.1111/1751-7915.12071

**Published:** 2013-08-06

**Authors:** Ken-ichi Lee, Naoki Kobayashi, Maiko Watanabe, Yoshiko Sugita-Konishi, Hirokazu Tsubone, Susumu Kumagai, Yukiko Hara-Kudo

**Affiliations:** 1 Graduate School of Agricultural and Life Sciences, the University of Tokyo1-1-1, Yayoi, Bunkyo-ku, Tokyo, 113-8657, Japan; 2 Division of Microbiology, National Institute of Health Sciences1-18-1, Kamiyoga, Setagaya-ku, Tokyo, 158-8501, Japan

## Abstract

To elucidate the effect of fungal hyphae on the behaviour of Shiga toxin-producing *Escherichia coli* (STEC) O157, the spread and change in stress resistance of the bacterium were evaluated after coculture with 11 species of food-related fungi including fermentation starters. Spread distances of STEC O157 varied depending on the co-cultured fungal species, and the motile bacterial strain spread for longer distances than the non-motile strain. The population of STEC O157 increased when co-cultured on colonies of nine fungal species but decreased on colonies of *Emericella nidulans* and *Aspergillus ochraceus*. Confocal scanning microscopy visualization of green fluorescent protein-tagged STEC O157 on fungal hyphae revealed that the bacterium colonized in the water film that existed on and between hyphae. To investigate the physiological changes in STEC O157 caused by co-culturing with fungi, the bacterium was harvested after 7 days of co-culturing and tested for acid resistance. After co-culture with eight fungal species, STEC O157 showed greater acid resistance compared to those cultured without fungi. Our results indicate that fungal hyphae can spread the contamination of STEC O157 and can also enhance the stress resistance of the bacteria.

## Introduction

Shiga toxin-producing *Escherichia coli* (STEC) are important cause of foodborne disease and often cause diarrhoea, haemorrhagic colitis and haemolytic uremic syndrome in humans (Su and Brandt, 1995; Gyles, 2007). About 70% of human cases with STEC infection in Japan were attributed to STEC O157 (National Institute of Infectious Diseases, 2011). Traditionally, STEC O157 was primarily associated with beef; however, recent outbreaks of STEC O157 have been associated with the consumption of cheese and fresh produce, which has raised concern that these products can be sources of STEC O157 infection (Erickson and Doyle, 2007; Franz and van Bruggen, 2008; Baylis, 2009).

In cheese and fresh produce, there are several commensal microorganisms derived from cattle and fermentation starters, as well as various microorganisms from the environment (Irlinger and Mounier, 2009). These coexisting microorganisms can interact with STEC O157 and may affect the behaviour of the pathogen. Interactions between pathogens and fungi are not well understood, although these interactions can play significant roles in the ecology of these microorganisms (Frey-Klett *et al*., 2011). Recently, results of several studies elucidated that various fungi enhance the growth and survival of bacterial pathogens in food model systems (Bevilacqua *et al*., 2008; Cibelli *et al*., 2008; Lee *et al*., 2012a). These growth and survival enhancements could be attributable to proteolysis and undefined metabolites derived from fungi.

Additionally, studies on soil and oral microorganisms revealed that fungal hyphae themselves affect bacterial behaviour (Bianciotto *et al*., 1996; Wargo and Hogan, 2006; Seneviratne *et al*., 2008; Nazir *et al*., 2010). Unlike bacteria, filamentous fungi can spread on and penetrate the surface of food and soil with ease. *Achromobacter*, *Bacillus* and *Pseudomonas* can attach to fungal hyphae and spread along the surface, which enable the bacteria to spread in the soil environment (Wong and Griffin, 1976; Bianciotto *et al*., 1996; Kohlmeier *et al*., 2005). Such physical interaction between bacteria and fungal hyphae play an important role in establishment of bacteria on plant roots and spread in soil. (Wong and Griffin, 1976; Bianciotto *et al*., 1996; Kohlmeier *et al*., 2005; Gurtler *et al*., 2013). However, the extent of physical interaction between bacteria and fungi remains unclear in the food environment, such as cheese and fresh produce. Therefore, the behaviour and physiological change of bacteria on fungal hyphae requires investigation.

In this study, to gain insight into the role played by fungal hyphae on the behaviour of STEC O157, the spread and growth of the bacterium on colonies of food-related fungi were investigated. The localization of STEC O157 on fungal hyphae was visualized by confocal microscopy of green fluorescent protein (GFP)-tagged STEC O157. Furthermore, stress resistance of STEC O157 to acid, after co-culture with fungi, was evaluated to assess physiological changes in the bacterium.

## Results and discussion

### Spread and growth of STEC O157 on fungal colonies

Spread and growth of STEC O157 on a fungal colony was evaluated by the method of Kohlmeier and colleagues (2005), with slight modifications. Briefly, fungal spores were inoculated at one end of a rectangular strip (width 40 × depth 10 × height 10 mm) of potato dextrose agar (PDA, Eiken Chemical, Tokyo, Japan), and the inoculated agar was incubated at 25°C. When the diameter of the fungal colony reached 20 mm, a motile or non-motile STEC O157 strain was inoculated at the same position where the fungus was inoculated. After incubation for 7 days at 25°C, the spread distance and the population of STEC O157 were assessed. As fermentation starters, *Geotrichum candidum*, *Penicillium camemberti*, *Penicillium nalgiovense* and *Penicillium roqueforti* were used. As food-spoilage fungi, *Alternaria alternata*, *Aspergillus ochraceus*, *Cladosporium sphaerospermum*, *Colletotrichum* sp., *Emericella nidulans*, *Fusarium oxysporum* and *Rhizopus* sp. were used (Table 1).

**Table 1:** Characteristics of bacterial and fungal strains used in this study

Strains	Origin	Motility^a^	Genotype
STEC O157			
ATCC43895	Meat	>45 mm day^−1^	*stx1 and stx2*
ESC138	Bovine faeces	–	*stx1 and stx2c*
Filamentous fungi			
*Alternaria alternata* TSY213	Unknown		
*Aspergillus ochraceus* TSY119	Unknown		
*Cladosporium sphaerospermum* TSY380	Hospital wall		
*Colletotrichum* sp. TSY208	Lemon		
*Emericella nidulans* TSY100	Horse bedding		
*Fusarium oxysporum* TSY0965	Unknown		
*Geotrichum candidum* C4-1	Cheese		
*Penicillium camemberti* C3-3	Cheese		
*Penicillium nalgiovense* M3-1	Sausage		
*Penicillium roqueforti* C10-1	Cheese		
*Rhizopus* sp. TSY79	Bedding		

a Motility of STEC O157 was measured by the method of Rashid and Kornberg (2000).

The spread-distance of STEC O157 varied among the fungal species used for co-culturing (Table 2). On colonies of *Rhizopus* sp., STEC O157 reached to the edge of the fungal colony regardless of the bacterial motility. On colonies of *G. candidum*, STEC O157 reached almost to the edge of the fungal colony regardless of the bacterial motility. However, on colonies of *A. alternata*, *C. sphaerospermum, Colletotrichum* sp. and *F. oxysporum*, the motile strain of STEC O157 spread over long distances (> 50% of the diameter of the fungal colony), while the non-motile strain spread over short distances (< 50% of the diameter of the fungal colony). On colonies of *P. camemberti* and *P. nalgiovense*, STEC O157 spread over short distances, regardless of the bacterial motility. Neither STEC O157 strain demonstrated any spread on colonies of *A. ochraceus*, *E. nidulans* or *P. roqueforti*. Meanwhile, the presence of bacteria did not apparently effect on the diameter of fungi.

**Table 2:** Spread-distance and the number of viable cells of STEC O157 on various fungal colonies and hydrophobicity of fungi

Fungal species	Day when the fungal colony diameter reached to 20 mm	Fungal colony diameter (mm; mean ± SD)	Hydrophobicity of fungi (%)		Maximum mobilization distance (mm; mean ± SD)		STEC O157 (log_10_CFU/strip; mean ± SD)	
			Outer	Inner	Motile	Non-motile	Motile	Non-motile
*Alternaria alternata*	6	40	17.5	15.0	40^a^	11 ± 6^a^	8.6 ± 0.4	8.3 ± 0.2
*Aspergillus ochraceus*	6	39 ± 2	67.5	62.5	0	0	<1	<1
*Cladosporium sphaerospermum*	17	33 ± 5	42.5	42.5	22 ± 8^a^	3 ± 1^a^	7.8 ± 0.6	7.6 ± 0.1
*Colletotrichum* sp.	4	40	17.5	2.5	35 ± 6^a^	5 ± 5^a^	8.6 ± 0.2^b^	8.1 ± 0.1^b^
*Emericella nidulans*	14	40	62.5	55.0	0	0	6.1 ± 0.2	5.5 ± 0.3
*Fusarium oxysporum*	5	40	45.0	57.5	31 ± 10^a^	4 ± 3^a^	8.1 ± 0.0^b^	7.7 ± 0.1^b^
*Geotrichum candidum*	6	36 ± 2	65.0	65.0	35 ± 2	29 ± 8	8.9 ± 0.1^b^	8.5 ± 0.0^b^
*Penicillium camemberti*	11	29 ± 4	65.0	92.5	5 ± 2	2 ± 2	8.2 ± 0.1^b^	7.8 ± 0.1^b^
*Penicillium nalgiovense*	14	33 ± 2	85.0	87.5	12 ± 12	10 ± 5	7.9 ± 0.1	7.9 ± 0.1
*Penicillium roqueforti*	8	38 ± 2	60.0	62.5	0	0	8.1 ± 0.1	8.1 ± 0.0
*Rhizopus* sp.	4	40	55.0	55.0	40	40	8.2 ± 0.2	8.1 ± 0.1

Mobilization and growth of STEC O157 on a fungal colony was evaluated by the method of Kohlmeier and colleagues (2005) with slight modifications. STEC O157 was incubated in tryptic soy broth (TSB; Becton, Dickinson and Company, New Jersey, USA) at 37°C for 20 h prior to use. Fungi were incubated on a PDA at 25°C for 2 weeks prior to use. A fungus was inoculated at the one end of a rectangular agar strip of width 40 × depth 10 × height 10 mm PDA and the agar strip was incubated at 25°C. When a diameter of the fungal colony reached to 20 mm, STEC O157 was inoculated at the same place of the where the fungus was inoculated. Inoculum size of the motile and non-motile strain was 6.8 ± 0.1 and 6.5 ± 0.1 log_10_CFU/strip respectively) After incubation of the agar strip for 7 days at 25°C, the agar strip was stamped onto a tryptone soya agar (TSA, Oxoid Ltd, Hampshire, UK) and incubated overnight at 37°C. The diameter of a bacterial colony was regarded as a distance of mobilization of STEC O157. The agar strip after the stamping was crushed in phosphate-buffered saline (PBS; Nissui Pharmaceutical Co., Ltd, Tokyo, Japan) and mixed at a full speed of an automatic mixer (S-100, Taitec Co., Ltd, Saitama, Japan). The suspension was serially diluted with PBS and pour-plated onto TSA. All plates were incubated at 37°C, and colonies were counted after 48 h.

The surface hydrophobicity of fungal colonies was measured using the alcohol percentage test (Chau *et al*., 2010). A series of aqueous ethanol solutions were prepared in 2.5% increments, from 0 to 100% ethanol. Four-microliter droplets of the ethanol solutions were applied to the surface of fungal colonies, and the time interval used for infiltration of the droplets was < 5 s. Replicates of three droplets on the inner and outer zone of a fungal colony were assessed. The minimum ethanol concentration that managed to infiltrate into a fungal colony was regarded as an indicator of the surface-hydrophobicity of the fungus, therefore, larger values represent higher hydrophobicity.

a Significant difference (*P* < 0.05) between STEC strains by Student's *t*-test.

b Significant difference (*P* < 0.05) between STEC strains by Student's *t*-test.

The STEC O157 population differed among fungi co-cultured (Table 2). On colonies of *A. alternata, Colletotrichum* sp., *C. sphaerospermum*, *F. oxysporum*, *G. candidum*, *P. camemberti*, *P. nalgiovense*, *P. roqueforti* and *Rhizopus* sp., the population of STEC O157 significantly (Student's *t* test; *P* < 0.05) increased from the inoculum size. On colonies of *E. nidulans*, the population of the STEC O157 decreased significantly (Student's *t* test; *P* < 0.05) from the inoculum size. On colonies of *A. ochraceus*, the population of STEC O157 decreased below the detection limit.

Based on the spread-distance and change in population of STEC O157, the fungi used in this study could be grouped as follows: on *Rhizopus* sp. and *G. candidum*, STEC O157 can spread over long distances and can grow regardless of the bacterial motility; on *A. alternata*, *C. sphaerospermum*, *Colletotrichum* sp. and *F. oxysporum*, the motile strain of STEC O157 can spread for longer distances than the non-motile strain, but both strains can grow; on *P. nalgiovense*, *P. camemberti* and *P. roqueforti*, the spread-distance of STEC O157 is impeded markedly, but the bacterium can still grow; on *A. ochraceus* and *E. nidulans*, STEC O157 cannot spread and grow.

In previous studies, it was reported that some motile bacteria spread on fungal colonies (Kohlmeier *et al*., 2005; Wick *et al*., 2007). The importance of bacterial motility for the spread on hyphae was also shown in our study. However, we found that non-motile bacteria can also spread on fungal colonies. Kohlmeier and colleagues (2005) showed that latex beads could not spread along fungal hyphae, therefore, the spread of the non-motile STEC O157 along fungal hyphae should be explained by biological factors. In our study, the population of STEC O157 increased on colonies of *A. alternata, Colletotrichum* sp., *C. sphaerospermum*, *F. oxysporum*, *G. candidum*, *P. camemberti*, *P. nalgiovense*, *P. roqueforti* and *Rhizopus* sp., regardless of the bacterial motility. The growth of STEC O157 could contribute to the spread of the non-motile strain. On fungal colonies, various amino acids and polysaccharides from dead hyphae and fungal exudates enable various bacteria to grow (Sun *et al*., 1999; Leveau and Preston, 2008; Warmink *et al*., 2009). However, the bacterial motility also affects the growth on fungal colonies. On the colony of *G. candidum*, *Colletotrichum* sp., *F. oxysporum* and *P. camemberti*, the bacterial population of the motile strain is significantly higher (*P* < 0.05, Student's *t*-test) than that of the non-motile strain. On these fungal colonies, motility might be important for exploring the favourable place to grow although the difference in genetic background between STEC O157 strains should be taken into consideration. On the other hand, the population of STEC O157 decreased on the colony of *A. ochraceus* and *E. nidulans*. Because these two species are phylogenetically closely related, a similar mechanism may inhibit the growth of STEC O157.

### Association of bacterial spread distance and hydrophobicity of fungal colonies

Fungal colonies on agar plate demonstrate two layers: an aerial mycelium layer on the surface and a biofilm layer underneath the aerial mycelium layer (Rahardjo and Rinzema, 2007). This aqueous biofilm layer would play an important role in the spread of bacteria. Previous studies showed that the low hydrophobicity of fungal hyphae allow bacteria to spread on surface (Wong and Griffin, 1976; Kohlmeier *et al*., 2005), because continuous water film on mycelia facilitate bacterial spread. Thus, to investigate the fungal factor that affects the bacterial spread, the hydrophobicity of the surface layer of fungal colonies were measured using the alcohol percentage test described by Chau and colleagues (2010) (Table 2). Firstly, the correlation between the hydrophobicity of fungi and the spread distance of STEC O157 was considered. An apparent correlation was not detected, according to the determination coefficient, R^2^, in either outer (motile strain, R^2^ = 0.38; non-motile strain, 0.00) and inner (motile, 0.32; non-motile, 0.00) zone of fungal colony. Secondly, we focused on the spread distance attributable to the bacterial motility. The spread distance in the non-motile strain of STEC O157 was subtracted from that of the motile strain in each fungus co-cultured. We assumed that these values represent the spread distance attributable to the bacterial motility. Then, R^2^ value was calculated between these R^2^ values and the hydrophobicity. For this calculation, data from *A. ocharaceus*, *E. nidulans*, *P. roqueforti* and *Rhizopus* sp. was excluded, because the spread distance attributable to the bacterial motility could not be calculated. On these fungi, both bacterial strains reached to the edge of the fungal colony or did not spread at all. Interestingly, R^2^ values showed a good correlation between the fungal hydrophobicity and the spread distance attributable to the bacterial motility (R^2^ = 0.86 in outer zone; 0.78 in inner zone). From these results, it is found that the motile strain of STEC O157 spread farther along hydrophilic fungal hyphae. Negative effect of fungal hydrophobicity on bacterial spread along hyphae is accordance to previous studies (Kohlmeier *et al*., 2005; Wick *et al*., 2007; Warmink and van Elsas, 2008), but we newly showed the effect between the motile and non-motile strain of the same bacterial species. On the other hand, variation in spread distances in the non-motile strain requires further investigation. One explanation for variation of the bacterial spread distances is the effect of fungal metabolites. The study of Wong and Griffin (1976) showed the spread of *Bacillus subtilis* along dead mycelia of *Pythium ultimum*, while the same fungus facilitate spread of pseudomonads in other studies (Leben, 1984; Wick *et al*., 2007). These studies indicate that fungal metabolites inhibit the growth or spread of some bacteria. In our results, metabolites from *A. ochraceus*, *E. nidulans* and *P. roqueforti* might inhibit the growth or spread of STEC O157. Hyphal growth can also facilitate the passive spread of the bacteria. Because *Rhizopus* sp. grows fast, the non-motile strain might be passively translocated along the hyphal growth. The bacterial growth can also affect the bacterial spread, because the bacterial population increased 100 to 1000 fold on most of fungi.

### Microscopic observation of STEC O157 on fungal hyphae

**Fig 1:**
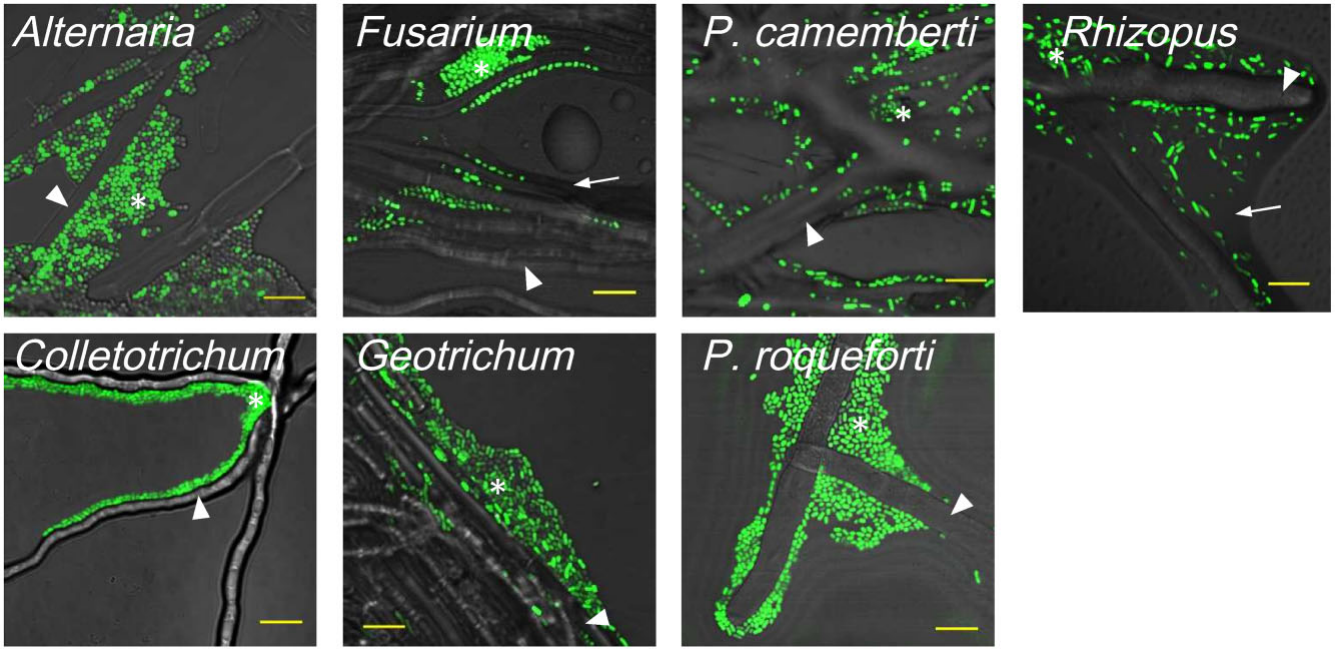
Confocal laser scanning microscopy analysis of various fungal hyphae and colonization on the hyphae by GFP-tagged motile strain of STEC O157.GFP-tagged STEC O157 was prepared by transformation of the motile and non-motile strains of STEC O157 with a GFP expression vector (pAcGFP1; Clontech Laboratories, Inc., Palo Alto, CA, USA) using the calcium chloride method (Sambrook and Russell, 2001). A fungus was inoculated on a cube-shaped TSA containing 100 μg ml^−1^ of ampicillin (Wako Pure Chemical Industries, Ltd, Osaka, Japan) in a 35 mm glass-based dish (a small Petri dish of which the bottom is made of cover glass; Asahi Techno Glass, Chiba, Japan) and incubated at 25°C. TSA was used rather than PDA as the glucose in PDA down-regulates the *lac* promoter of pAcGFP1 and subsequently inhibits the expression of GFP. After the fungal colony reached the bottom of the glass-based dish, GFP-tagged STEC O157 was inoculated in the same position where the fungus was inoculated. The inoculated agar was co-cultured at 25°C, and GFP-tagged STEC O157 on the hyphae was observed daily up to day 7 by using a confocal laser microscope (FV1000-D, Olympus Corporation, Tokyo, Japan) with an oil immersion objective lens (UplanApo 100 ×, Olympus) and FLUOVIEW software (Olympus). The excitation and emission wavelength for GFP was 488 and 510 nm respectively. GFP-tagged STEC O157 appears as green cells. Although some cells do not appear as green due to the variation in GFP expression, all bacilli in the pictures are STEC O157. The mycelia and GFP-tagged STEC O157 were observed on agar-glass interface. In this region, both biofilm layer and aerial mycelia layer can be observed. Fungal hyphae and the water film on the hyphae are indicated by arrow heads and arrows respectively. Asterisks show the biofilm-like structure formed by STEC O157. Bar = 8 μm.

To visualize the STEC O157 on fungal colonies, GFP-tagged STEC O157 was co-cultured with fungi and was observed using a confocal scanning microscope (Fig. 1). The motile strain of STEC O157 was observed mainly in the liquid layer (water film) that was formed on and between fungal hyphae of *A. alternata*, *Colletotrichum* sp., *F. oxysporum*, *G. candidum*, *P. camemberti*, *P. roqueforti* and *Rhizopus* sp. (arrow in Fig. 1). In the water film, planktonic and biofilm state of the motile strain was observed. The planktonic cells were swimming in the water film. Typical water film and swimming STEC O157 was shown in the image of *Rhizopus* sp. in Fig. 1. STEC O157 colonized and formed a biofilm-like structure on fungal hyphae where the mycelia were dense (asterisks in Fig. 1). The motile strain of STEC O157 was not observed when it was co-cultured with *A. ochraceus*, *C. sphaerospermum*, *E. nidulans* and *P. nalgiovense* (data not shown). On the colony of *P. nalgiovense* and *C. sphaerospermum*, STEC O157 was not observed, although the bacterium spread in the experiment using agar strip. These fungi showed characteristic traits in the experiment using the agar strip and the hydrophobicity test. The population of STEC O157 on these fungi were relatively low compared to other fungi. In addition, the hydrophobicity of *P. nalgiovense* was the highest in the fungi used. In the experiment using the glass-base dish, interaction of bacteria and fungi can be seen where the fungal mycelia are sparse. In this region, the bacterial population and high hydrophobicity of the fungi might heavily affect the bacterial spread.

The localization of the non-motile strain was similar to that of the motile strain but was not observed swimming in the water film (data not shown). The non-motile strain was not observed either when it was co-cultured with *A. ochraceus*, *C. sphaerospermum*, *E. nidulans* and *P. nalgiovense*. In addition to them, the bacterium was not observed when it was co-cultured with *F. oxysporum*, *P. camemberti* and *P. roqueforti*.

Our results are consistent with the studies of Kohlmeier and colleagues (2005) and Furuno and colleagues (2010) that showed that continuous water films formed along fungal hyphae could facilitate the spread of motile bacteria, in addition to passive translocation of bacteria upon fungal growth. The amount of fungal exudates may affect the thickness of the water film and the surface hydrophobicity of hyphae. Developing accurate quantification methods for the amount of exudate and surface hydrophobicity of hyphae would be required to explain the variation in spread distance of bacteria among fungal species. In addition to the water film, Warmink and colleagues (2011) suggested that bacterial biofilm formation on fungal hyphae is likely to be involved in facilitating spread. Because the non-motile strains of STEC O157 formed a biofilm-like structure in the same manner as the motile strain, biofilm formation may be involved in facilitating spread of the non-motile strain.

### Stress resistance of STEC O157 after co-culture with fungi

**Fig 2:**
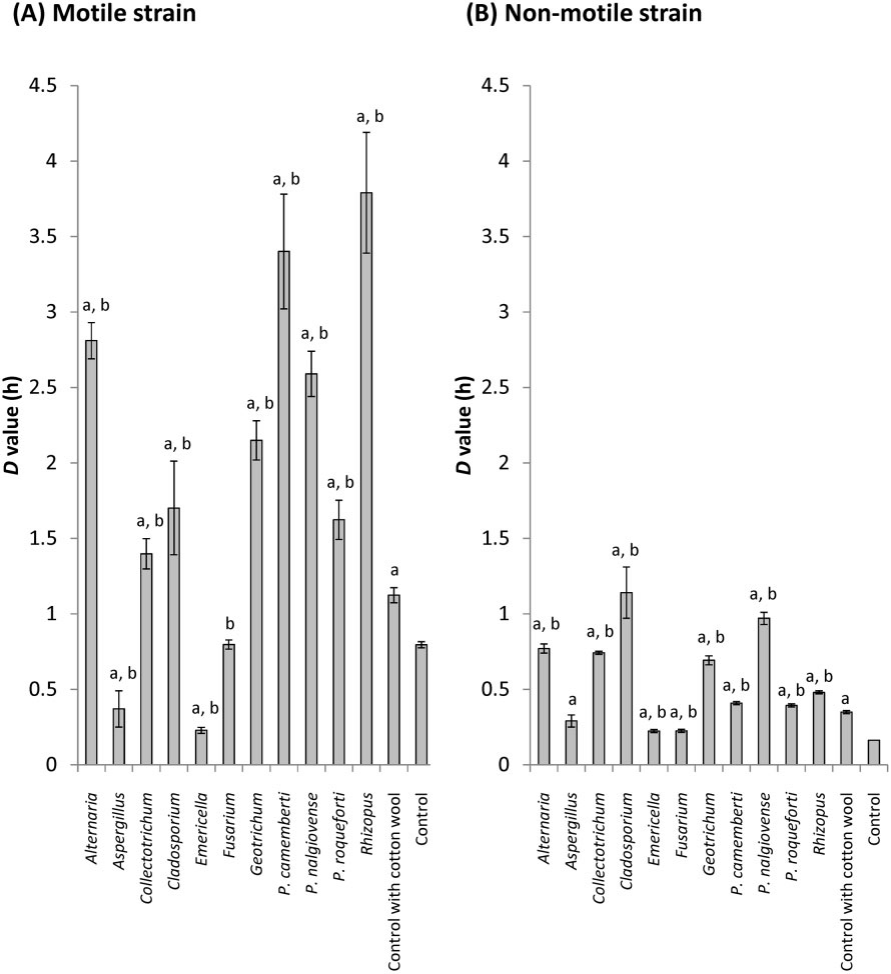
*D* values at pH 2.5 of (A) motile and (B) non-motile strains of STEC O157 after co-culture with various fungi.Approximately 10^8^ CFU of motile or non-motile STEC O157 was inoculated onto a 7-day-old fungal colony grown on PDA. As control, the same amount of STEC O157 was inoculated onto PDA without fungi. In addition, to investigate the effect of a filamentous structure *per se* on the stress resistance of STEC O157, the bacterium was inoculated onto a sterilized rectangular cotton wool (width 20 × depth 40 × height 4 mm) that was placed onto PDA as ‘control with cotton wool’. After 7-day incubation at 25°C, the agar strip was crushed and suspended in PBS. The suspension was mixed at a full speed by using an automatic mixer and centrifuged at 4000 × g for 10 min. The supernatant was removed, and the pellet washed twice with PBS. The pellet was re-suspended again with PBS and was diluted 100-fold into 10 ml of EG medium acidified with hydrogen chloride at pH 2.5. EG medium is commonly used in evaluating acid resistance of *E. coli* (Lin *et al*., 1996). The broth was incubated at 37°C, and the population of STEC O157 was measured at 0, 1, 2 and 4 h. To enumerate STEC O157, the inoculated broth was serially diluted with PBS and pour-plated onto TSA. After incubation for 48 h at 37°C, colonies were counted. *D* values were calculated using the formula (*D* value = −1/slope), where slope represents the linear regression of the data including all the sampling points. The R^2^ values of the linear regression analyses were more than 0.8 in every analysis. The experiments were performed in triplicate. Error bars represent the standard deviation of the three trials. Each letter in the figures represents a significant difference by Student's *t*-test as follows:A. *P* < 0.05 compared to the control.B. *P* < 0.05 compared to the control with cotton wool.

A biofilm-like structure of STEC O157 on fungal hyphae was observed under microscopic observation (shown as asterisks in Fig. 1). In various bacterial species, biofilm growth affected their stress resistance (Dykes *et al*., 2003; Kubota *et al*., 2009). Therefore, stress resistance of STEC O157 after co-culture with fungi was investigated using an acid resistance assay. Acid stress was chosen as STEC O157 must survive in the acidic gastric fluid for causing infections in humans. Briefly, after co-culture with fungi for 7 days at 25°C, STEC O157 was collected from the colony and inoculated into minimal E glucose medium (EG medium) (Vogel and Bonner, 1956), acidified with hydrogen chloride (Kanto Chemical Co., Inc., Tokyo, Japan) at pH 2.5. As an indicator for acid stress resistance in STEC O157, decimal reduction time (*D* value) was used. *D* value is the time required at a certain environment, such as heat and osmotic pressure, to decrease 90% of the organisms and is commonly used to explore appropriate control measures to a pathogen. (Barkley and Richardson, 1994) The larger values mean greater resistance of the bacteria used. In our study, the motile strain had greater resistance than the non-motile strain (Fig. 2). Variation in acid resistance among strains of STEC O157 has been known (Lee *et al*., 2012b), and these strains would have genetic difference in acid resistance. *D* values of both the motile and non-motile STEC O157 strains co-cultured with *A. alternata*, *Colletotrichum* sp., *C. sphaerospermum*, *G. candidum*, *P. camemberti*, *P. nalgiovense*, *P. roqueforti* and *Rhizopus* sp. were significantly higher (Student's *t* test; *P* < 0.01) than those of the control, which is monoculture of STEC O157 on PDA (Fig. 2). In contrast, *D* values of the motile strain of STEC O157 after co-culture with *A. ochraceus* and *E. nidulans* were significantly lower (Student's *t* test; *P* < 0.01) than that of the control. Co-culture with *F. oxysporum* did not affect the *D* value of the motile strain; however, in the case of non-motile strain of STEC O157, *D* values after co-culture with *A. ochraceus*, *E. nidulans* and *F. oxysporum* were significantly higher (Student's *t* test; *P* < 0.01).

Interestingly, incubation of STEC O157 in cotton wool on PDA (shown as control with cotton wool in Fig. 2) increased the *D* value. Cotton wool was used to evaluate the abiotic effect of fungal hyphae on the stress resistance of STEC O157. From these results, it was clear that a complex fibre network itself affects the stress resistance of STEC O157. In a fibre network, it is assumed that bacteria can form biofilm structure. Previously, close relationship between biofilm formation and stress resistance has been reported (Shanks *et al*., 2007; Zhang *et al*., 2007). Therefore, STEC O157 that exists as biofilm states could confer resistance against acid. However, *D* values after co-culture with most of the fungi were significantly higher (Student's *t* test; *P* < 0.05) than those of the control cultured with cotton wool. Therefore, both biotic and abiotic fungal factors alter the stress resistance of coexisting bacteria. Previously, Gawande and Bhagwat (2002) also showed that incubation of *Salmonella* on polyethersulfone membranes and tissue paper enhanced bacterial stress resistance and the change in stress resistance required protein synthesis. Because several genes that mediate oxidative stress and heat shock responses were induced upon the growth of *E. coli* on surfaces, such as agar plates (Cuny *et al*., 2007), these regulons may contribute to changes in stress resistance.

In addition to the abiotic effects of fungal hyphae, biotic effects could enhance the stress resistance of STEC O157. Previously, several studies showed that spent cultures of fungi enhance the growth and survival of pathogenic bacteria (Bevilacqua *et al*., 2008; Cibelli *et al*., 2008; Lee *et al*., 2012a). These substances may facilitate STEC biofilm formation, and subsequently enhance stress resistance.

Moreover, the attachment apparatus would play an important role during interaction with fungal hyphae. Warmink and van Elsas (2008) reported that bacteria with a type three secretion system (TTSS) can successfully attach to fungal hyphae. TTSS contribute to biofilm formation (Moreira *et al*., 2006) and virulence (Coburn *et al*., 2007), in addition to attachment to eukaryotic cells (Shaw *et al*., 2008). Further investigation of the relationships between TTSS and spread of bacteria on fungal hyphae, which may affect bacterial virulence, is necessary.

In conclusion, our results demonstrated that filamentous fungi can facilitate the spread and growth of STEC O157 on fungal colonies. Moreover, after co-culture with fungi, the stress resistance of STEC O157 in an acid environment was enhanced compared to that of bacteria grown in the absence of fungi. Because food-related fungi enhanced the spread and stress resistance of STEC O157, the risk of infection with this bacterium in food with the growth of fungi may be quite different from food without fungi.
